# Understanding the NG2 Glial Scar after Spinal Cord Injury

**DOI:** 10.3389/fneur.2016.00199

**Published:** 2016-11-15

**Authors:** Amber R. Hackett, Jae K. Lee

**Affiliations:** ^1^Miami Project to Cure Paralysis, The Neuroscience Graduate Program, Department of Neurological Surgery, University of Miami Miller School of Medicine, Miami, FL, USA

**Keywords:** OPCs, oligodendrocytes, scar formation, astroglial scar, oligodendrocyte progenitor cells, axon regeneration

## Abstract

NG2 cells, also known as oligodendrocyte progenitor cells, are located throughout the central nervous system and serve as a pool of progenitors to differentiate into oligodendrocytes. In response to spinal cord injury (SCI), NG2 cells increase their proliferation and differentiation into remyelinating oligodendrocytes. While astrocytes are typically associated with being the major cell type in the glial scar, many NG2 cells also accumulate within the glial scar but their function remains poorly understood. Similar to astrocytes, these cells hypertrophy, upregulate expression of chondroitin sulfate proteoglycans, inhibit axon regeneration, contribute to the glial-fibrotic scar border, and some even differentiate into astrocytes. Whether NG2 cells also have a role in other astrocyte functions, such as preventing the spread of infiltrating leukocytes and expression of inflammatory cytokines, is not yet known. Thus, NG2 cells are not only important for remyelination after SCI but are also a major component of the glial scar with functions that overlap with astrocytes in this region. In this review, we describe the signaling pathways important for the proliferation and differentiation of NG2 cells, as well as the role of NG2 cells in scar formation and tissue repair.

## Introduction

Many oligodendrocytes are lost after contusive spinal cord injury (SCI) (1, 2), leaving axons demyelinated and impairing proper conduction of action potentials (3–6). Although new remyelinating oligodendrocytes are formed after SCI (7–11), normal levels of myelination are not achieved (6). Pre-existing oligodendrocytes do not contribute to remyelination (12); however, NG2 cells, also known as oligodendrocyte progenitor cells (OPCs), are ubiquitously distributed throughout the central nervous system (CNS) and are capable of differentiating into oligodendrocytes in the adult CNS (13). Thus, targeting their proliferation and differentiation is an appealing target to promote remyelination after CNS injury. NG2 cells are present in increased numbers surrounding the lesion site (2, 7, 8, 14), and many studies have investigated the mechanisms underlying their differentiation into oligodendrocytes and their contribution to remyelination (15–17).

However, a large number of NG2 cells that do not differentiate into oligodendrocytes are present within the glial scar, which has been traditionally synonymous with reactive astrocytes. Interestingly, similar to astrocytes, these NG2 cells hypertrophy and upregulate expression of chondroitin sulfate proteoglycans (CSPGs) after CNS injury (18). In fact, NG2 is itself a CSPG (gene name is *cspg4*) and can inhibit axon growth *in vitro* (19). Interestingly, NG2 cells have the capacity to differentiate into astrocytes at the CNS injury site, as discussed in more detail below. Thus, NG2 cells are potentially a major contributor to the axon regeneration inhibition by the glial scar.

In addition to their role in axon growth inhibition, NG2 cells may share other properties with astrocytes. For example, astrocytes play an important role in preventing the spread of infiltrating leukocytes, and their ablation leads to increased neuron and oligodendrocyte loss (20, 21). Astrocytes also play a major role in the immune response after contusive SCI through secretion of pro-inflammatory cytokines and chemokines (22, 23). In this review article, we will discuss methods of investigating NG2 cells in the context of SCI, the mechanisms underlying the proliferation of NG2 cells after SCI, as well as their contribution to the glial scar including axon regeneration, wound healing and inflammation.

## Scar Formation after Contusive SCI

**Figure 1:**
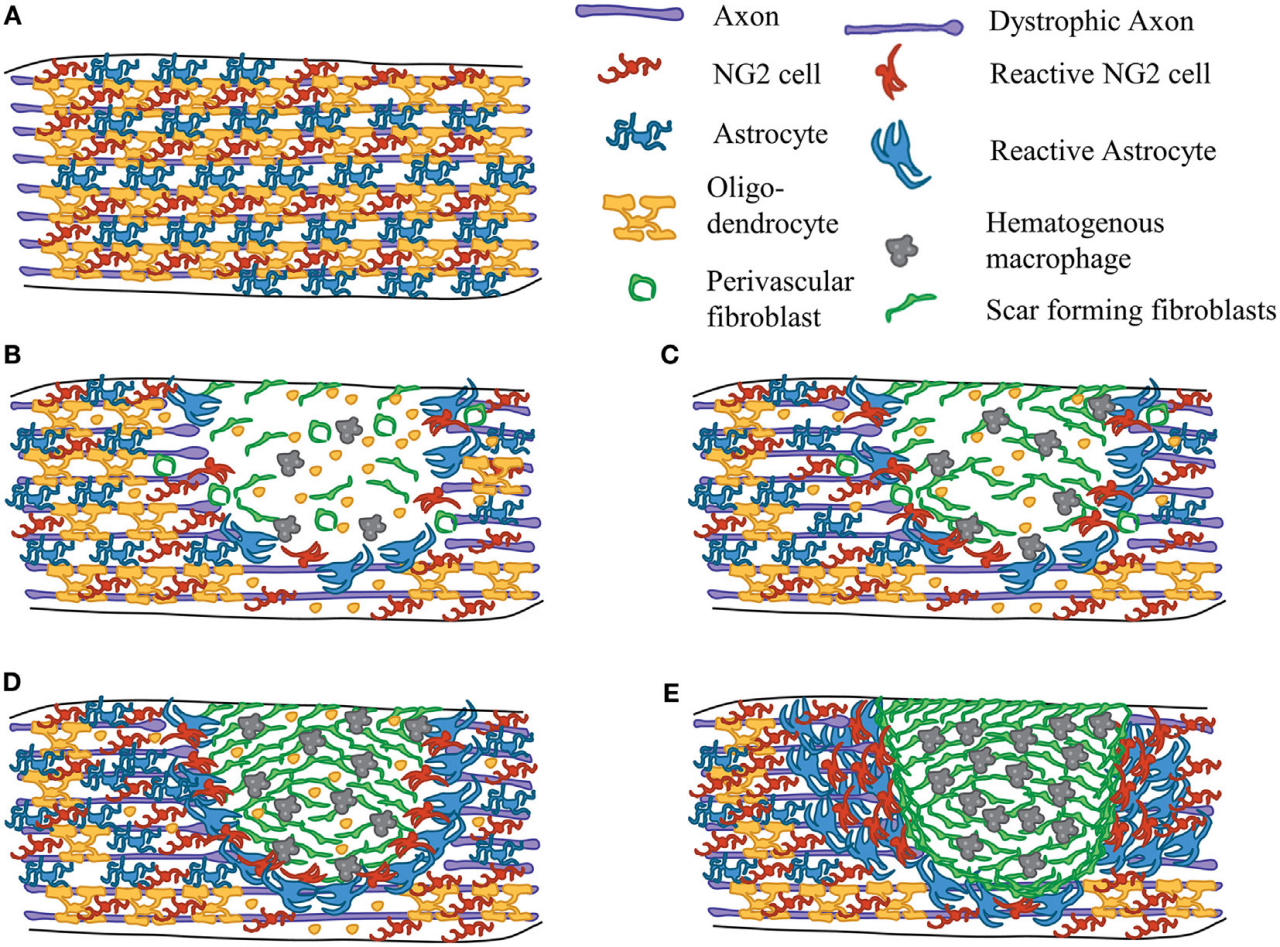
**Scar formation after SCI**. Diagram depicting the events of scar formation after contusive SCI in mice. Astrocytes (blue), NG2 cells (red), and myelinating oligodendrocytes (yellow) in the uninjured spinal cord white matter **(A)**. Early after SCI, cell death occurs within the lesion site and axons are damaged. Microglia (not shown) and astrocytes respond by secreting cytokines and chemokines. NG2 cells react and proliferate around the lesion site. Macrophages (gray) begin to infiltrate the lesion core and perivascular fibroblasts (green) begin to delaminate from blood vessels **(B)**. Inflammation causes secondary death of oligodendrocytes and neurons leading to accumulation of myelin debris in the injury site **(C)**. Macrophage and fibroblast density peaks at 7 days after SCI **(D)**. By 2 weeks after SCI, the scar has matured. There are tight borders between the fibrotic scar (consisting of fibroblasts and macrophages) and the glial scar (consisting of astrocytes, NG2 cells, and microglia) **(E)**. The relative number of cells may not accurately reflect actual *in vivo* pathology.

Figure 1 depicts a diagram of the cellular reactions after contusive SCI in mice. Differences between mice, rat, and human SCI will be addressed where appropriate. In the uninjured spinal cord, astrocytes, oligodendrocytes, and NG2 cells are located throughout the parenchyma (Figure 1A). Contusive SCI leads to large scale death of neurons and glia at the site of injury, shearing of ascending and descending axons, and damage to the vasculature. This damage leads to large-scale hemorrhage at the site of the lesion, which leads to the release of factors that contribute to the immune response, and responses from resident glia (24, 25). Microglia reacts within hours after injury by accumulating around the lesion site and secreting pro-inflammatory cytokines and chemokines that which contribute to the immune response (26). While NG2 cells have been shown to proliferate and migrate short distances toward the lesion site after laser induced injury (27), their migration capacity has not been investigated in more clinically relevant traumatic injuries. Astrocytes also proliferate, hypertrophy, and upregulate expression of glial fibrillary acidic protein (GFAP), and secrete cytokines, chemokines, growth factors, and CSPGs (28). Increased inflammation leads to secondary damage to neurons and oligodendrocytes, as well as axonal dieback characterized by dystrophic endings (1, 29) (Figures 1B–D). Myelin debris and CSPGs, both inhibitory to axon regeneration, accumulate in the lesion core and the glial scar. Hematogenous macrophages start to infiltrate the lesion (30, 31) and attract perivascular fibroblasts that separate from blood vessels and form the fibrotic scar (32, 33) peaking in density by 7 days after SCI. By 14 days after SCI, the scar has started to mature and form tight borders between the glial and fibrotic components of the scar (20, 21, 33) (Figure 1E). (At around this time in rats and humans, a fluid-filled cavity starts to form in parts of the fibrotic scar, whereas in mice, the fibrotic scar contracts slightly over time.) The formation of this scar is dependent on the interactions between CNS cells, namely microglia, NG2 cells, and astrocytes, with non-CNS cells, namely hematogenous macrophages and fibroblasts. In human SCI, astrocytes and NG2 cells were readily detected in the glial scar, and macrophages in the lesion core, within days after SCI (34). Understanding their individual contributions to scar formation is essential for designing both regenerative and neuroprotective therapies for SCI. In this review, we will focus primarily on the role of NG2 cells in the context of the glial scar formation after SCI.

## NG2 Cell Fate Mapping Strategies after CNS Injury

Proper understanding of NG2 cells after SCI requires proper understanding of the tools that have been used to study them, namely antibodies and transgenic mouse lines. In the uninjured brain and spinal cord, antibodies against NG2 and PDGFRα (platelet-derived growth factor receptor alpha) label NG2^+^ glia, but NG2 antibodies also label pericytes that express this CSPG (35–37) (Table 1). After CNS injury, NG2 expression is upregulated at the injury site (18), but many cells including pericytes, non-myelinating Schwann cells, and macrophages also express NG2 (38, 39) (Table 1), making the use of the NG2 antibody alone insufficient to definitively identify NG2^+^ glial cells. Similarly, PDGFRα antibodies can label fibroblasts, rather than NG2 glia, at the injury site (39) (Table 1). Since cells that are NG2^+^ macrophages, NG2^+^ pericytes, or PDGFRα^+^ fibroblasts are often counted as NG2 glia, the use of these antibodies as sole markers for NG2 glia has led to the misconception that NG2 glia are located within the injury core (GFAP-negative region) and has mostly likely contributed to the highly variable reports of NG2 cell density across different studies (40). For the remainder of this review, our use of NG2 cells refers to NG2^+^ glia (and not pericytes), and we use these two terms along with OPC interchangeably. Antibodies against Olig2 has also been used to identify NG2 cells, but they also label mature oligodendrocytes, and several reports have shown that a small population of protoplasmic astrocytes (10, 41) and reactive astrocytes can also express the transcription factor Olig2 after CNS injury (42, 43) (Table 1). Thus, co-labeling with NG2 and Olig2 antibodies may be the best method of histologically detecting NG2 cells after SCI.

**Table 1:** **Antibodies used to label NG2 cells after SCI**.

Antibody	NG2 glia	Pericytes	Astrocytes	OLs	Macrophages	Schwann cells
**Uninjured**						
NG2 (39)	+	+				
PDGFRα (39)	+	+				
Olig2 (42, 43)	+			+		
**Injured**						
NG2 (39)	+	+			+	+
PDGFRα (39)	+	+				
Olig2 (42, 43)	+		+	+		

Prior to the advent of genetic fate mapping using transgenic mice expressing cell type-specific Cre recombinase, several attempts to understand the fate of NG2 cells after SCI were made. One of the first attempts to study the fate of NG2 cells after SCI utilized a Mahoney retrovirus with reporter expression driven by the NG2 promoter (9). Injection of this virus into the injury site-labeled dividing NG2 cells, however, due to the fact that it was administered after SCI, it also labeled a large number of macrophages (since some of them upregulate NG2 as discussed above) (39, 44). This study also reported that a high percentage of NG2 cells differentiate into GFAP^+^ astrocytes (35–50%); however, this could have included astrocytes that upregulated NG2 after SCI. Shortly after, Lytle et al. (43) used the CNP-EGFP mice (which labels 2′,3′-cyclic-nucleotide 3′-phosphodiesterase^+^ NG2 cells and oligodendrocytes) to determine the response of NG2 cells after contusive SCI and reported that a large population of NG2^+^ cells were EGFP^−^. This could have been due to CNPase only being expressed in NG2 cells that have already committed to the oligodendrocyte lineage since CNPase is expressed later than PDGFRα and NG2 during development (45, 46). However, it is also possible that scar forming NG2 cells downregulate CNPase expression after injury. Together, these results suggest that NG2 glia comprise both myelinating cells as well as non-myelinating, scar forming cells after contusive SCI.

Transgenic mice expressing tamoxifen-inducible Cre under cell-specific promoters (Cre-ER mice) have been particularly useful for studying fate of NG2 cells after SCI. Although PDGFRα-CreER (13, 47), NG2-CreER (48), and Olig2-CreER (41) mice have been used extensively to either fate map and/or conditionally delete genes in NG2 cells, each mouse line has its advantages and disadvantages. The NG2-CreER mouse line has a recombination efficiency of about 30–40% of NG2 cells (48, 49), while the PDGFRα-CreER has a recombination efficiency of over 90% (47). Low recombination efficiency is often desirable for lineage tracing studies, while high recombination efficiency is often desirable for functional studies. Similar to the limitations of antibodies as discussed above, these transgenic lines label cells other than NG2 glia. The NG2-CreER mice label pericytes (49, 50) whereas the PDGFRα-CreER mice label fibroblasts at the injury site (unpublished observations), and the Olig2-CreER mice label oligodendrocytes as well as a small population of astrocytes (10, 51). Although we did not observe contributions of NG2^+^ pericytes to scar formation after SCI in mice, it is possible that experimental manipulations (drugs, viruses, or genes deletions) could induce them to contribute to scar formation (49). Thus, these off-target labeling must be carefully considered when interpreting any NG2 cell genetic fate mapping studies involving these mouse lines.

One possible solution to circumvent these technical hurdles is to combine genetic labeling of NG2 cells with antibody co-labeling. Genetically labeled cells in NG2-CreER mice can be co-labeled with Olig2 or PDGFRβ antibody to distinguish NG2 cells from pericytes/fibroblasts respectively. Alternatively, instead of using Rosa26 reporter mice, Olig2 promoter-driven reporter mice can be used in combination with NG2-CreER or PDGFRα-CreER mice, which would label NG2 glia without labeling pericytes. This Olig2 strategy can be used to express not only fluorescent reporters but also proteins such as diphtheria toxin receptor (52) that can be used to probe the function of NG2 cells more specifically. However, such Olig2 reporter mice have not yet been reported in the literature.

## Proliferation and Oligodendrogenesis

NG2 cells have been shown to react to CNS injuries such as traumatic brain injury (18), demyelination (53), and contusive SCI (2). This response is reminiscent of astrocyte reactivity as they surround the lesion site and hypertrophy (18). Whereas NG2 cells are normally evenly dispersed throughout the spinal cord (13) and maintain territories due to the dynamic filopodia being repulsed by neighboring NG2 cells (27), their processes become intertwined as they form the glial scar. Two-photon live imaging has revealed NG2 cells react to laser injury by migrating only short distances toward the lesion (27), suggesting that the large number of NG2 cells at the injury site is most likely due to local proliferation rather than migration. In fact, the percentage of proliferating NG2 cells is increased sixfold (2) and NG2 cells comprise nearly one half of bromodeoxyuridine (BrdU)-labeled cells, 3 days after SCI (7). This is most likely an underestimate since it does not account for NG2 cells that differentiated into oligodendrocytes and/or astrocytes after injury. Overall, these data suggest that NG2 cells have a significant capacity to proliferate after SCI.

As NG2 cells differentiate into oligodendrocytes, they lose expression of the NG2 antigen. NG2 cells are capable of differentiating directly into oligodendrocytes without cell division (27), but they often differentiate after division, where one or both differentiate into oligodendrocytes within 6–8 days. This represents a critical window where their fate after proliferation can be determined by the microenvironment of the injury site (48, 54). For example, myelin damage can accelerate and promote NG2 cell differentiation into oligodendrocytes (54). Sensory deprivation induced by whisker clipping can reduce oligodendrogenesis after NG2 cell division, suggesting that neuronal activity promotes differentiation of NG2 cells into oligodendrocytes (54). Conversely, optogenetic stimulation of neurons can increase the proliferation of NG2 cells and their subsequent differentiation into oligodendrocytes (55). This raises the possibility that the myelin and neuronal damage after SCI may create an environment that significantly influences NG2 cell differentiation.

Several factors important for proliferation and differentiation of NG2 cells are upregulated after SCI. These include fibroblast growth factor 2 (FGF2) (56, 57), glial growth factor 2 (GGF2) (58, 59), and Wnts (60). FGF2 is a potent mitogen for NG2 cells *in vitro* (56). Deletion of FGFR1 and FGFR2 in NG2 cells reduces oligodendrogenesis and remyelination chronically after cuprizone-induced demyelination (61). FGF2 is increased for at least a month after SCI (57) and intraspinal injection of FGF2 (62) was shown to improve functional recovery after SCI. GGF has been shown to increase the proliferation of NG2 cells while inhibiting their differentiation *in vitro* (63). Subcutaneous injection of GGF2 increases NG2 cell proliferation, oligodendrogenesis, and functional recovery after SCI (59) as well as increased functional recovery and myelination after experimental autoimmune encephalomyelitis (EAE) (64). Wnts have been shown to play a major role in proliferation of NG2 cells during development (65) and are upregulated after SCI (60). Overexpression of activated β-catenin, a downstream mediator of Wnt signaling, results in developmental hypomyelination and delayed remyelination after demyelination (65). Wnt3A-conditioned media increases proliferation of NG2 cells *in vitro* and deletion of β-catenin in NG2 cells leads to reduced proliferation of NG2 in the glial scar after contusive SCI (66).

Cytokines such as ciliary neurotrophic factor (CNTF), leukemia inhibitory factor (LIF), and tumor necrosis factor (TNF) are also important in the proliferation and differentiation of NG2 cells (67). CNTF and LIF promote oligodendrocyte differentiation *in vitro* (67), however, CNTF knockout (68) and LIF knockout mice (69) only have a developmental delay in oligodendrogenesis, indicating that these factors may be a mediator of oligodendrogenesis early in development. Daily intraperitoneal administration of CNTF increases numbers of NG2 cells, oligodendrocytes, and neurons and improves outcome after EAE (70). Deletion of the transcription factor signal transducer and activator of transcription 3 (STAT3), which is downstream of CNTF, LIF, and several other cytokines, delays oligodendrogenesis without affecting proliferation after SCI (49). Accordingly, overexpression of a constitutively active STAT3 using an adenovirus leads to increased oligodendrocyte differentiation *in vitro* (71). Deletion of suppressor of cytokine signaling 3 (SOCS3), a negative regulator of STAT3, leads to enhanced proliferation of NG2 cells in the glial scar but does not affect their differentiation after SCI, suggesting a non-canonical STAT3/SOCS3 signaling mechanism in NG2 cells after SCI (49). Although the proinflammatory cytokine TNFα is typically associated with oligodendrocyte death, it may have important roles in NG2 cell response to injury and subsequent remyelination. TNFα signaling through TNFR2 is important for NG2 cell proliferation and differentiation after cuprizone-induced demyelination (72). Similarly, genetic deletion of TNFR2 using the CNPase-Cre mouse resulted in impaired functional recovery, reduced number of NG2 cells, and impaired oligodendrocyte differentiation and remyelination after EAE (73). While similar genetic studies have not been performed after SCI, pharmacological blockade of TNFR1, but not TNFR2, promotes functional recovery SCI (74).

## NG2 Cell Lineage Plasticity after Injury

### Astrogliogenesis

Both NG2 cells and astrocytes are derived from radial glia during development (75, 76). In addition, NG2 cells isolated *in vitro* differentiate into astrocytes as well as oligodendrocytes (77). Thus, while NG2 cells differentiate only into oligodendrocytes in the normal CNS, these observations provide a mechanistic basis for the potential of NG2 cells to differentiate into astrocytes in the injured CNS. The advent of Cre-loxP technology has allowed rigorous testing of NG2 cell lineage plasticity *in vivo*. The NG2-Cre mouse line revealed that, indeed, a population of NG2 cells could differentiate into protoplasmic astrocytes in the ventrolateral forebrain gray matter (78) and spinal cord (79) during development. As mentioned above, Sellers et al. injected Mahoney retrovirus with a reporter driven by the NG2 promoter into the injured spinal cord, and found that 35–54% of reporter-labeled cells were GFAP^+^ astrocytes (limitations of this technique is discussed above).

Using CreER mice to permanently label a population of NG2 cells prior to injury has led to similar results. The NG2-CreER and PDGFRα-CreER mouse lines both revealed that NG2 cells can only differentiate into oligodendrocytes in the uninjured adult CNS (13, 47, 48). To determine if NG2 cells had lineage plasticity after injury, the Olig2-CreER mice were used in cortical stab injury (41) and dorsal hemisection SCI (10). However, due to the fact that 5% of labeled cells were GFAP^+^ in the uninjured condition, it was difficult to determine if NG2 cells had astroglial fate. Using the NG2-CreER mice in which astrocytes are not labeled in the uninjured spinal cord, 8% of NG2 cells expressed GFAP at 10 days post cortical stab injury (80). Since the percentage of reporter-labeled cells that co-localized with GFAP decreased to 2% by 30 days after injury and many cells retained NG2 expression, it has been suggested that these NG2 cells transiently express GFAP after injury and that NG2 cell-derived astrocytes are not major contributors to the astroglial scar (80). However, after contusive SCI where inflammation, secondary damage, and astrogliosis is much greater than stab wounds, 25% of reporter-labeled cells in the NG2-CreER mice expressed GFAP at 1 week after SCI and 8% by 4 weeks after injury (49).

Possible mechanisms by which NG2 cells differentiate into astrocytes after SCI could be similar to the mechanisms underlying astrogliogenesis during development. These include the Janus kinase (JAK)/STAT3 (81), bone morphogenetic protein (BMP) (82), and/or Olig2 signaling pathways (83, 84). BMP2 and BMP4 are known to promote astrogliogenesis from NG2 cells *in vitro* (85). Both BMP2 and BMP4 are upregulated after SCI (86), and intraspinal injection of BMP4 leads to increased differentiation of transplanted NG2 progenitors into GFAP^+^ astrocytes (9). When NG2 cells are treated with conditioned media from reactive astrocytes isolated from injured spinal cords, it reduces their differentiation into O1^+^ oligodendrocytes and increases their expression of GFAP (87), suggesting that the injured spinal cord could provide a niche for NG2 cell differentiation into astrocytes. Astrocytes isolated from the injured spinal cord have increased expression of BMP2/BMP4 compared to uninjured spinal cord astrocytes and BMP2 is increased in reactive astrocyte conditioned media, suggesting that astrocytes are a major source of BMPs after SCI (87). NG2 cells increase expression of the BMP downstream effector Smad after exposure to reactive astrocyte-conditioned media with an associated decrease in MBP and increase in GFAP expression, which is reversed upon treatment with the BMP inhibitor noggin (87). Together, these data suggest that BMPs may be derived from reactive astrocytes and promote NG2 cell differentiation into astrocytes after contusive SCI.

In addition to BMPs, the JAK-STAT3 signaling pathway could also be important in astrogliogenesis from NG2 cells after CNS injury. The JAK-STAT3 signaling pathway is important for astrocyte differentiation from nestin^+^ cortical precursor cells and STAT3 binds to the GFAP promoter (81, 88). In addition, developmental astrogliogenesis is impaired in LIF knockout mice and gp130 knockout mice (89, 90). However, neither deletion of STAT3 nor its negative regulator SOCS3 significantly affects NG2 cell differentiation into astrocytes after SCI (49). Overexpression of the oligodendrocyte transcription factor Olig2 reduced astrocyte differentiation from neural stem cells *in vitro* (83) while deletion of Olig2 in developing NG2 cells leads to increased astrocyte production at the expense of oligodendrogenesis and myelination (91). However, genetic deletion of Olig2 does not affect astrogliogenesis from NG2 cells after cortical stab injury (80). Together, these data suggest that NG2 cells might differentiate into astrocytes by a mechanism different from developmental processes.

### Differentiation into Schwann Cells

After contusive SCI, there are many Schwann cells at the injury site (92, 93). Since there are no Schwann cells in the normal spinal cord and since the majority of the myelin protein 0 (P0^+^) myelinating Schwann cells are located in the dorsal column after SCI, it was thought that these Schwann cells had migrated from the dorsal roots. However, genetic lineage tracing revealed that, after focal demyelination in the dorsal column white matter, the majority of Schwann cells were derived from NG2 cells (94). This is also supported by a recent study in which a dorsal rhizotomy did not lead to a significant decrease in Schwann cells at the SCI site, indicating that the peripheral nervous system (PNS) is not a major source of Schwann cells present at the injury site (95). While there is accumulating evidence that NG2 cells can differentiate into Schwann cells after SCI, there are several issues that need to be carefully considered. First, in addition to dorsal roots, the ventral roots as well as nerve fibers on blood vessels may also serve as sources of Schwann cells (96). Second, unlike the ability of NG2 cells to differentiate into astrocytes *in vitro*, there have been no reports of NG2 cells differentiating into Schwann cells *in vitro*. Last, whereas NG2 cells and astrocytes are derived from the neural tube, Schwann cells are derived from the neural crest, thereby making the mechanism by which NG2 cells differentiate into Schwann cells ontogenetically more complex than the mechanism of their differentiation into astrocytes.

## Contributions to Axon Regeneration

Increased CSPG expression is widely considered to be a major inhibitory barrier to axon regeneration after CNS injury. Phosphocan, neurocan, versican, and brevican are all upregulated after SCI (97). While reactive astrocytes are considered a major source of CSPGs (98), NG2 cells have also been shown to secrete versican and neurocan *in vitro* (99–101). Unlike other CSPGs, NG2 is typically expressed on the cell membrane rather than as a secreted factor. However, its extracellular domain can be shed from the cell surface *via* cleavage by metalloproteinases (MMPs) (102). Increased expression of NG2 in the glial scar and its ability to inhibit neurite outgrowth *in vitro* indicate that NG2 cells may be major inhibitors of axon regeneration (19). The NG2 proteoglycan leads to inhibition of cerebellar granule neuron neurite outgrowth even after digestion with chrondroitinase ABC (ChABC), indicating that it is not just the glycosaminoglycan (GAG) side chains but also the core proteoglycan that is inhibitory to axon growth (19). Treatment with intraspinal injection of NG2 neutralizing antibody leads to enhanced regeneration of ascending sensory axons after SCI (103), and long-term delivery of NG2 neutralizing antibody through an osmotic pump improves conduction and functional recovery after SCI (104). Together, these studies suggest that NG2 proteoglycan is inhibitory to axon regeneration.

However, the inhibitory properties of NG2 proteoglycan does not necessarily mean that NG2 cells themselves are inhibitory. Despite the increased levels of NG2, several studies have noted that NG2 cells are often associated with regenerating axons, and similar findings have been reported for astrocytes (105–107). Neonatal hippocampal neurites grow better on NG2 cells than on poly-l-lysine and laminin (PLL), even after overexpressing NG2 using an adenovirus (108). In addition, regenerating axons are observed more frequently in areas of the spinal cord that are NG2^+^ after SCI (39, 109) and may facilitate axon entry into Schwann cell grafts after SCI (110). Also, *cspg4* knockout mice display less serotonergic axons that are able to cross into the lesion (111), as well as increased dieback of sensory axons after SCI (112). In addition, regenerating dorsal root ganglion (DRG) axons associate with NG2-expressing cells after dorsal column crush (112). These data suggest that while NG2 proteoglycan may inhibit axon regeneration, NG2 cells themselves may be permissive to axon growth (108, 112). This is similar to the role of reactive astrocytes where even though their expression of CSPG is inhibitory to axon regeneration, reactive astrocytes themselves may be permissive to, and even necessary for, axon regeneration (105–107). Thus, we must be cautious in classifying cells as inhibitory to axon regeneration based solely on their expression of CSPGs.

While axons may be able to use NG2 cells as a growth-permissive substrate, the fact that regenerating axons can form terminal synaptic contacts with NG2 cells implies that the net effect prevents axonal growth (112–114). The ability of NG2 cells to form synapse with axons has been known for quite some time (115), but its significance in the context of axon regeneration is just beginning to be appreciated. NG2 cells form “synaptic-like” structures with DRG axons *in vitro* (112), and NG2 cells are associated with dystrophic sensory axons (116). Furthermore, there is presynaptic differentiation of injured sensory axons along the CNS/PNS border after dorsal root crush injury (117). Together, these data indicate that NG2 cells may inhibit axon regeneration by both expression of the inhibitory NG2 proteoglycan as well as formation of synaptic contacts.

## Contributions to Inflammation

Astrocytes contribute to the inflammatory response after CNS injury and attenuating their expression of proinflammatory cytokines and chemokines leads to improved functional outcome (118–123). While astrocytes and microglia have been the focus of neuroinflammatory studies, there is accumulating evidence that NG2 cells may also contribute to the inflammatory response. Genetic deletion of Act1, an activator of NFκB (nuclear factor kappa-light-chain-enhancer of activated B cells) *via* interleukin-17 (IL-17) signaling, in NG2 cells, leads to reduced expression of proinflammatory chemokines, reduced leukocyte infiltration, and improved functional outcome after EAE (124). Upon stimulation *in vitro*, NG2 cells increase expression of multiple proinflammatory chemokines and cytokines as well as MMPs (124, 125). In addition, NG2 cells upregulate IL1β and C-C motif chemokine ligand 2 (CCL2) after cuprizone demyelination (126). Interestingly, deletion of β-catenin in NG2 cells leads to reduced Iba1^+^ macrophage/microglia density around the lesion after SCI and also reduced astrogliosis, suggesting that NG2 cells may play a role in attracting macrophages after CNS injury (66). Therefore, these studies raise the possibility that NG2 cells may be a major contributor to inflammation after CNS injury, and future studies need to directly address this possibility, especially in the context of SCI.

## Summary

**Figure 2:**
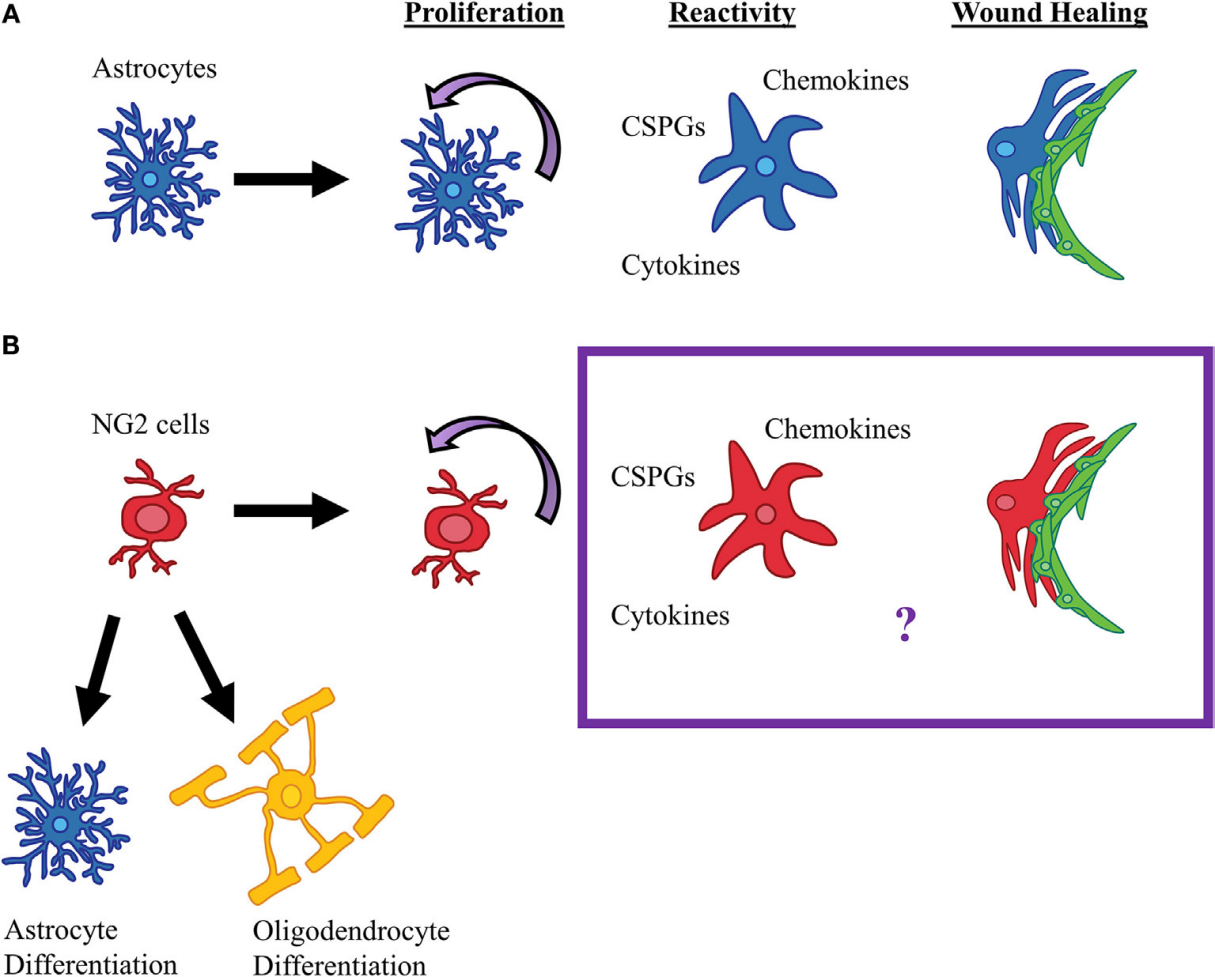
**Reactive gliosis after SCI**. **(A)** After SCI in mice and rats, astrocytes proliferate; secrete cytokines, chemokines, and CSPGs; and form glial-fibrotic borders. **(B)** It is known that NG2 cells proliferate, differentiate into oligodendrocytes and astrocytes, and contribute to scar formation after SCI, however, whether NG2 cells contribute to the glial scar by secreting cytokines or contribute to the wound healing process is currently unknown.

The glial scar has been synonymous with reactive astrocytes, but there is substantial evidence indicating that NG2 cells are also a major part of the glial scar, both physically and functionally. Similar to astrocytes, NG2 cells react to SCI by proliferating, becoming hypertrophic, and upregulating CSPG expression (Figure 2). However, unlike astrocytes, NG2 cells can differentiate into other cell types, namely oligodendrocytes, astrocytes, and perhaps even Schwann cells. This lineage plasticity of NG2 cells raise the possibility that they can be targeted to promote endogenous repair of the injured spinal cord. Most NG2 cells remain undifferentiated in the glial scar region, and these NG2 cells contribute to axon regeneration failure by expressing CSPGs and forming synaptic structures that prevent further axonal growth. Similar to astrocytes, NG2 cells may also contribute to neuroinflammation, which remains an area that has been underappreciated in the field (Figure 2). Therefore, NG2 cells share similarities and differences with astrocytes as a part of the glial scar, which present novel mechanisms that may be targeted to promote repair after SCI.

## Author Contributions

AH and JL selected the content and wrote the manuscript.

## Conflict of Interest Statement

The authors declare that the research was conducted in the absence of any commercial or financial relationships that could be construed as a potential conflict of interest.
